# A Bi-Spectral Microbolometer Sensor for Wildfire Measurement

**DOI:** 10.3390/s21113690

**Published:** 2021-05-26

**Authors:** Denis Dufour, Loïc Le Noc, Bruno Tremblay, Mathieu N. Tremblay, Francis Généreux, Marc Terroux, Carl Vachon, Melanie J. Wheatley, Joshua M. Johnston, Mike Wotton, Patrice Topart

**Affiliations:** 1 INO (Institut National d’Optique), 2740 Einstein Street, Québec, QC G1P 4S4, Canada; Loic.Le.Noc@ino.ca (L.L.N.); Bruno.Tremblay@ino.ca (B.T.); MathieuN.Tremblay@ino.ca (M.N.T.); Francis.Genereux@ino.ca (F.G.); Marc.Terroux@ino.ca (M.T.); Carl.Vachon@ino.ca (C.V.); Patrice.Topart@ino.ca (P.T.); 2 Faculty of Forestry, University of Toronto, 33 Willcocks Street, Toronto, ON M5S 3B3, Canada; melanie.wheatley@mail.utoronto.ca; 3 Canadian Forest Service, Great Lakes Forestry Centre, 1219 Queen St. E., Sault Ste. Marie, ON P6A 2E5, Canada; Joshua.johnston@canada.ca (J.M.J.); mike.wotton@canada.ca (M.W.)

**Keywords:** wildfire, microbolometer, FRP, radiometric, UAV, satellite

## Abstract

This study describes the development of a prototype bi-spectral microbolometer sensor system designed explicitly for radiometric measurement and characterization of wildfire mid- and long-wave infrared radiances. The system is tested experimentally over moderate-scale experimental burns coincident with FLIR reference imagery. Statistical comparison of the fire radiative power (FRP; W) retrievals suggest that this novel system is highly reliable for use in collecting radiometric measurements of biomass burning. As such, this study provides clear experimental evidence that mid-wave infrared microbolometers are capable of collecting FRP measurements. Furthermore, given the low resource nature of this detector type, it presents a suitable option for monitoring wildfire behaviour from low resource platforms such as unmanned aerial vehicles (UAVs) or nanosats.

## 1. Introduction

Globally, wildfires burn nearly 4.3 million km^2^ annually [1], with climate driving and controlling fire regimes [2]. However, anthropogenic burning (regardless of intent) is the dominant cause of wildfire ignition the world over [3,4,5]. Global wildfire activity is increasing under climate change [6,7]. This trend is worsened by continued population growth and the expansion of wildland urban interface across many regions of the world [8,9]. Not surprisingly, there has also been an increased frequency of catastrophic wildfire events in recent years (e.g., Australia, 2009, 2019/20; California, 2018; South America, 2019; the Mediterranean/Greece, 2018; the Arctic, 2019; Canada, 2016, 2017, 2018).

Emerging technologies may provide a means of supporting increasingly complex operations resulting from the growing threat of wildfires. Of particular interest has been the use of new remote sensing technologies, tools and information systems in wildfire operations [10]. Regionally, some countries implement Earth observation (EO) monitoring systems (e.g., Brazil, Canada, USA, South Africa), while the Global Wildfire Information System (GWIS) provides global EO fire monitoring services. Near real-time smoke monitoring derived from these EO products is available both regionally [11,12] and globally [13].

In an effort to expand the scientific and operational value of infrared (IR) imaging, there is a growing body of evidence suggesting that it can be used to characterize wildfire behaviour under experimental conditions. Specifically, through the automation of fire structure detection [14,15] and temporal analysis, it is possible to map and measure the rate and direction of spread [16,17,18], arguably with higher precision than traditional methods [19]. Additionally, the fire radiative power (FRP; W) [20] emitted from actively spreading flame fronts has been shown to be a strong predictor of fire line intensity (kW m^−1^) [21,22].

The potential utility of infrared remote sensing in the operational management of wildfires has spurred numerous technology developments. The government of Canada recently announced its intention to build the world’s first dedicated operational wildfire monitoring satellite, WildFireSat [23]. The technology behind this system has been proposed for smaller-scale applications [24]. In the airborne domain, there has also been substantial interest in the expanded use of unmanned aerial vehicles (UAV) in wildfire operations [25,26,27,28]. However, UAV applications are highly sensitive to payload mass and power requirements, which presents varying challenges in terms of integrating the type of IR systems necessary for comprehensive wildfire monitoring.

An ideal means to achieve infrared (IR) band radiometric imaging measurement in a small, lightweight and low-power system suitable for resource-constrained platforms such as UAVs is to use uncooled microbolometer sensors. Although initially primarily of interest for military applications, today, microbolometer sensors have become much less expensive to fabricate than cooled infrared detectors and are finding more uses in commercial applications. Notably, Institut national d’optique (INO) built the microbolometer sensors for the new infrared sensor technology (NIRST) instrument, which was launched on NASA’s SACD-Aquarius satellite in 2011 [29] and was a foundational step in the evolution of the WildFireSat mission. A dual-camera system operating in both the mid-wave infrared (MWIR) and long-wave infrared (LWIR) bands, NIRST was a pioneering demonstration of the use of microbolometer technology for wildfire monitoring from space, using a 512 × 3 format focal plane array. Since then, INO has been developing other microbolometer array formats for custom applications, such as the INO-384 detector, which has 384 × 288 pixels of 35 µm pitch [30]. The INO-384 focal plane array (FPA), in combination with the development of novel broadband absorber technologies such as gold black and resonant structures, has been used for nearly 10 years in a variety of leading-edge systems developed at INO, such as see-through Terahertz systems and hyperspectral imagers [31,32].

Notably, the wide infrared wavelength band sensitivity made possible by INO’s gold black absorber technology, combined with the recent availability of wide infrared band optics, led INO to develop the Microxcam-384-MLWIR camera, which offers uniform sensitivity in the 3 to 14 µm spectral range in a compact, lightweight and low power format. By fitting this camera with appropriate bandpass filters and a calibration system, it is possible to adapt it for wildfire measurement. Its low weight and power demand makes it ideal for UAV platforms.

In this study we describe the development of a novel bi-spectral microbolometer-based thermal imaging system test bench, based on the Microxcam-384-MLWIR camera, known as Bomberos. The suitability of Bomberos for wildfire monitoring applications is evaluated through a statistical comparison of its MWIR and LWIR bands to reference imagery collected simultaneously with a commercial photovoltaic detector-based MWIR radiometric imager (FLIR SC6703 camera) over a series of small-scale experimental biomass fires. Specifically, we compare the similarity of FRP measurement between the Bomberos MWIR and FLIR MWIR bands, and between the Bomberos MWIR and LWIR bands. In doing so, we demonstrate that Bomberos is an effective means of accurately characterizing wildfire energy, and present the very first field-based, observational evidence that MWIR microbolometers are capable of measuring FRP.

## 2. Materials and Methods

### 2.1. Bomberos

#### 2.1.1. System Description

**Figure 1:**
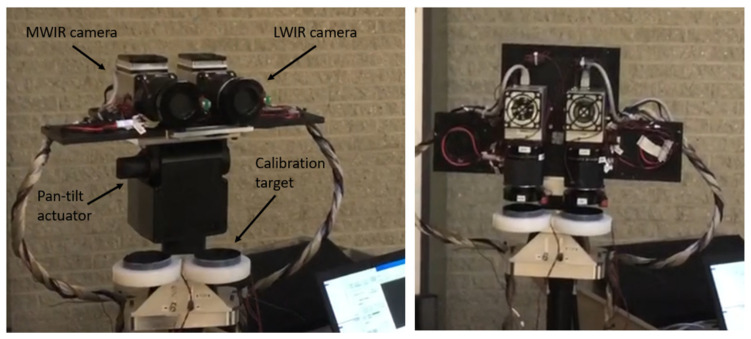
The Bomberos bi-spectral imaging system, shown in observation (left) and calibration (right) modes.

Bomberos was devised as a test bench to evaluate the performance of INO’s broadband microbolometer technology in FRP measurement. It consists of: (1) two infrared cameras mounted on a motorized pan-tilt actuator, (2) two blackbody calibration targets, (3) a thermal control system, (4) a visible-band camera for reference, (5) a tripod, and (6) two laptop computers for data acquisition. Photos of Bomberos are shown in Figure 1.

It should be noted that a potential future implementation for UAVs would be far lighter and more compact. Indeed, the very wide IR sensitivity of the INO gold black bolometers could make it possible to have a single camera, with butcher-block filters near the focal plane, to cover both the MWIR and LWIR bands. Such a camera would consume far less power than a cooled infrared camera. Indeed, the INO Microxcam-384-MLWIR camera core typically draws less than three Watts of power, excluding the detector’s thermo-electric cooler (TEC) that is used for temperature stabilization and which typically consumes one additional Watt. A single embedded computer would suffice to acquire imagery from such a bi-spectral camera on a UAV. Moreover, the motorized pan-tilt actuator plus blackbody plate assembly could be replaced by a single lightweight mechanical shutter with a high emissivity surface of known, measured temperature for calibration.

On the Bomberos system, the MWIR and LWIR cameras are modified INO Microxcam-384-MLWIR cameras that are identical aside from the bandpass filters and aperture masks. They both have a compact (61 × 61 × 65 mm, 360 g) Microxcam camera core, containing 3 to 14 µm gold-black coated 384 × 288 microbolometer detectors, and are configured with identical commercial broadband IR optics (Ophir SupIR radiometric lens model #65119, with F/1 aperture and 35 mm focal length). The Microxcam core contains a TEC element under the detector to maintain its temperature to a stable value (typically 25 °C). Each Microxcam core interfaces to a data acquisition and control computer via a Gig-E communication link and is powered by a 12 VDC supply. The two IR cameras are synchronized to measure the same scene at the same time.

Wavelength selectivity is provided by bandpass MWIR (3.4 to 4.2 µm) and LWIR (10.4 to 12.3 µm) one-inch diameter filter elements mounted on custom-designed filter holders between the sensor and the imaging lens. The wideband detectors can be quite sensitive to small temperature variations of these filters, particularly the MWIR filter, which is emissive in the LWIR range. Thereby, the filter and detector environment is temperature-controlled to 27 °C using TEC elements and temperature monitoring feedback. As described in more detail later, custom-designed aperture-limiting masks are added to the ends of the lenses to prevent excessive irradiance from reaching the detectors when exposed to high-temperature fires. Finally, mechanical shutters are installed just outside the lenses, to protect the detectors from excessive thermal signals (remanence effects, as described in the next section) when not in image acquisition mode.

The two cameras are mounted on pan-tilt actuators that can be used to scan different viewing angles, and to provide occasional downward-looking views of the blackbody calibration targets. These targets serve as a known temperature reference that enable converting detector signal intensity to scene radiance and apparent temperature. The targets consist of aluminum plates covered with high-emissivity paint (black velvet). The target temperatures are measured since these temperature values are required to apply radiometric correction, as described in the next sub-section. The targets are thermally passive, and therefore their temperature can vary depending on the ambient temperature.

The cameras, pan-tilt actuators and blackbody targets are all installed on a ruggedized tripod mount. A commercial small visible-band video camera was also installed next to the two infrared cameras, to provide contextual information for data analysis.

#### 2.1.2. Calibration and Measurement Procedure

Wildfire measurement requires the infrared cameras to be configured appropriately, in particular because of the large amount of power incident on the pixels when viewing a high temperature event. This can cause two problems: detector saturation and remanence. For the purposes of the Bomberos test campaign described later in this article, we assumed the maximum apparent fire temperature to be 900 °C. This is based on the previously measured intensity of similar experimental fires and the ground-projected pixel size. Under normal operation settings, the Bomberos camera signals (in particular the MWIR band one) would saturate well below this point. A simple way to avoid saturation is to reduce the bolometer integration time to reduce the responsivity. However, reducing the responsivity has the effect of reducing the pixel sensitivity, which is defined either as noise-equivalent spectral radiance (NESR) or noise-equivalent temperature difference (NETD). To counter this, the Bomberos cameras were configured to acquire images in a high dynamic range (HDR) mode with two alternating integration times, one to optimize the sensitivity for low temperature scenery, and the other to allow unsaturated fire temperature measurements up to 900 °C. The image frames with the two integration times are taken in rapid succession, 20 ms apart. The change in the scene radiance is assumed to be minimal within this time interval. Similar HDR sampling approaches have been used for FRP measurements elsewhere (e.g., [22]) and are used in the FLIR reference system used in this study (Section 2.2.2). Reconstructed images are then obtained by taking the high sensitivity/low dynamic range image and replacing saturated portions with image data from the low sensitivity/high dynamic range image.

As for the remanence effect, this phenomenon occurs when the VOx material in the bolometer is heated beyond a critical temperature point, for example, as a result of excessive incident irradiance on the pixels. The result is an extra offset signal on the bolometer that gradually goes away with time (on the order of several hours, but can be up to several days in severe cases), plus a loss of responsivity that also gradually returns to normal over time. The remanence effect was characterized in laboratory measurements by exposing the Bomberos cameras to various calibrated hot blackbody temperatures for different lengths of time. These measurements allowed the determination of the incident irradiance threshold on the pixels at which the remanence effect starts to occur. Based on these results, aperture-limiting circular masks were placed in front of the two cameras to restrict the amount of incident irradiance when the detector is exposed to the highest foreseen scene radiance (corresponding to 900 °C temperature exposure for 1 s every minute) to just below the remanence irradiance threshold. For the MWIR camera, a mask with a 6.5-mm hole was used, and for the LWIR camera, a 20-mm hole. Given the 35-mm diameter of the lenses, this reduces the effective lens aperture from F/1 to F/5.38 (MWIR) and F/1.75 (LWIR). Since the masks are in the field of view of the lenses, their thermal emission will contribute to the detector signal and must therefore be compensated for. It should be noted that these masks will reduce the sensitivity to low temperature scenery measurement, by a factor proportional to the square of the F-number.

To enable accurate radiometric measurements with a thermal imaging camera, all the factors that contribute to the signal measured on the detector must be considered. These can be expressed as in the following equation, where S is the digitized signal measured by a detector pixel (counts), R is the pixel responsivity (in counts/W), and O is the pixel offset signal (in counts):

(1) $$S\left(T_{scene}\right)=R\;\cdot \;\left\{\int_{\lambda =0}^{\infty }\left(G_{mask}\left(\lambda \right)\cdot L(\lambda ,T_{mask}\right)+G_{env}\left(\lambda \right)\cdot L\left(\lambda ,T_{env}\right)+G_{scene}\left(\lambda \right)\cdot L\left(\lambda ,T_{scene}\right)\;)\;d\lambda \;\right\}+O$$

The integral terms express that the power incident on a pixel is the sum of the thermal spectral radiances (*L(λ,T)*, representing Planck’s blackbody function as a function of wavelength and temperature from: (1) the scene being imaged (*T_scene_*), (2) the environment around the detector (*T_env_*), and (3) the aperture-limiting mask (*T_mask_*). The wavelength-dependent factors, *G(λ)* in the equation, are thermal-radiance-to-incident-power weighting factors for the different thermal contributors, defined by the camera geometry and optical properties (e.g., spectral emissivity and reflectance of the different components). To achieve accurate measurement of the scene radiance, all the other contributors to the signal should be removed. This can be achieved by performing an offset measurement close in time to the scene measurement, such that the detector environment and aperture mask temperatures are unchanged and can thus be eliminated by subtraction. The offset measurement consists of viewing the reference passive temperature targets, which are at a known temperature *T_offset_*. The resulting signal is as follows:

(2) $$S\left(T_{offset}\right)=R\;\cdot \;\left\{\int_{\lambda =0}^{\infty }\left(G_{mask}\left(\lambda \right)\cdot L(\lambda ,T_{mask}\right)+G_{env}\left(\lambda \right)\cdot L\left(\lambda ,T_{env}\right)+G_{scene}\left(\lambda \right)\cdot L\left(\lambda ,T_{offset}\right)\;)\;d\lambda \;\right\}+O$$

Subtracting the offset signal from the scene signal and rearranging the terms, we obtain the following expression for the scene radiance:

(3) $$L_{scene,band}=\frac{S\left(T_{scene}\right)-S\left(T_{offset}\right)}{R\cdot G_{scene,band}}+L_{offset,band}$$

where it was approximated that the *G(λ)* transfer function is uniform across the narrow wavelength range of the MWIR or LWIR filter through which the scene is viewed, such that the integration can be approximated as multiplication by a constant, i.e.,

(4) $$\int_{\lambda =0}^{\infty }G_{scene}\left(\lambda \right)\cdot L\left(\lambda ,T_{scene}\right)\;d\lambda \;\approx G_{scene,band}\cdot L_{scene,band}$$

This approximation was confirmed by measurements that showed that the detector signal *S* is indeed a linear function of the scene radiance *L*.

To summarize, the scene radiance can be determined by: (1) measuring the scene and offset detector signals, (2) calculating the in-band radiance *L_offset,band_* of the passive blackbody target of known temperature used for the offset measurement, and (3) determining the value of *R⋅G_scene,band_* for the given detector pixel.

The *R⋅G_scene,band_* values for each pixel and HDR setting were determined by laboratory measurement of calibrated laboratory blackbody sources by the Bomberos cameras as the blackbodies were set to a range of temperatures from 20 °C to 900 °C. Caution was taken to ensure the hot blackbody was not heating the cameras and mask during the measurements, which would cause an additional signal drift, inducing calibration errors. Accordingly, a small (one-inch diameter) high-temperature cavity blackbody, which provides a limited field of view to the camera and thereby limits heat transfer to the cameras, was used for the higher temperature measurements (above 300 °C). The calibration measurements were obtained at a range of pan and tilt settings to scan the entire field of view.

The Bomberos camera image acquisition and pan-tilt actuator control procedure was fully automated to provide the most accurate and repeatable measurements possible. A measurement cycle was set to last 60 s, and has the following steps:

t = 0 s: tilt the cameras downwards towards the calibration targets, then open the shutters every 3 s for a duration of 1 s each and acquire offset images.
t = 25 s: close the shutters and orient the cameras towards the scene of interest.
t = 29 s: open the shutters, acquire 1 s of scene images, then close the shutters.
t = 35 s: tilt the cameras downwards towards the calibration targets, then open the shutters every 3 s for a duration of 1 s each and acquire offset images.
t = 60 s: repeat the cycle.

As can be seen, the cameras spend most of their time with the shutters closed and looking at the calibration targets to minimize remanence effects. However, in future developments of its microbolometer-based fire sensors, INO plans to use their new high-resolution 1024 × 768 array, which is expected to exhibit a lesser remanence effect, and therefore can be operated for longer periods with the shutter open. Additionally, the remanence effect would be significantly less in a push-broom type scanning configuration, where the hot fire image would pass rapidly across the pixels, in contrast to this experiment where the cameras were staring at a hot fire at very close range.

### 2.2. Experimental Design and Protocol

#### 2.2.1. Layout

**Figure 2:**
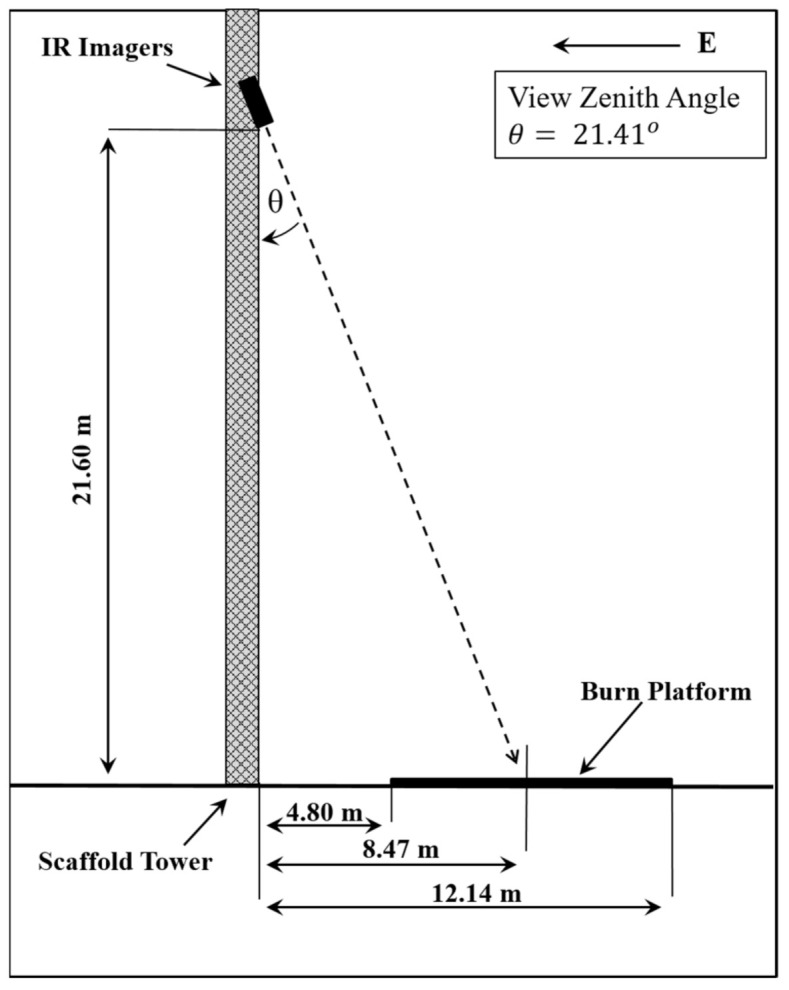
Visualization of the position of the Bomberos and FLIR SC6703 systems on the scaffold tower relative to the burn platform (not to scale).

A series of experimental burns was conducted for this study at the Canadian Forest Service’s Rose Experimental Burn Station [19,22] over three days in September of 2019. The Bomberos system was mounted ~21.6 m up a scaffold tower 4.8 m east of the burn platform. A commercial MWIR camera was mounted on the same level of the tower to serve as reference imagery for comparison. The view zenith angle for the imagers was ~21 degrees, as shown in Figure 2.

#### 2.2.2. Reference Imagery

A FLIR SC6703 with a narrow pass 3.9 µm spectral filter was used as the baseline MWIR reference point. The narrow spectral band and wide dynamic range (~423 to 1123 K) of this camera (achieved through HDR sampling of three integration times) has been proven to provide highly reliable FRP measurements [22]. In this study, it was configured to collect imagery at a frame rate of 30 Hz.

#### 2.2.3. Data Collection Protocol

Prior to ignition, the FLIR camera collected several frames in ambient imaging mode. This was to provide spatial reference points prior to collecting data in the higher temperature settings, which omit ambient temperatures. Once this was complete, the reference imager was placed in high-temperature mode, and image collection began at a rate of 30 Hz. At the same time, the Bomberos cameras were set to acquire continuously, as described in Section 2.1.2. The Bomberos cameras measured low-temperature background and high-temperature fires simultaneously due to the HDR acquisition mode.

**Figure 3:**
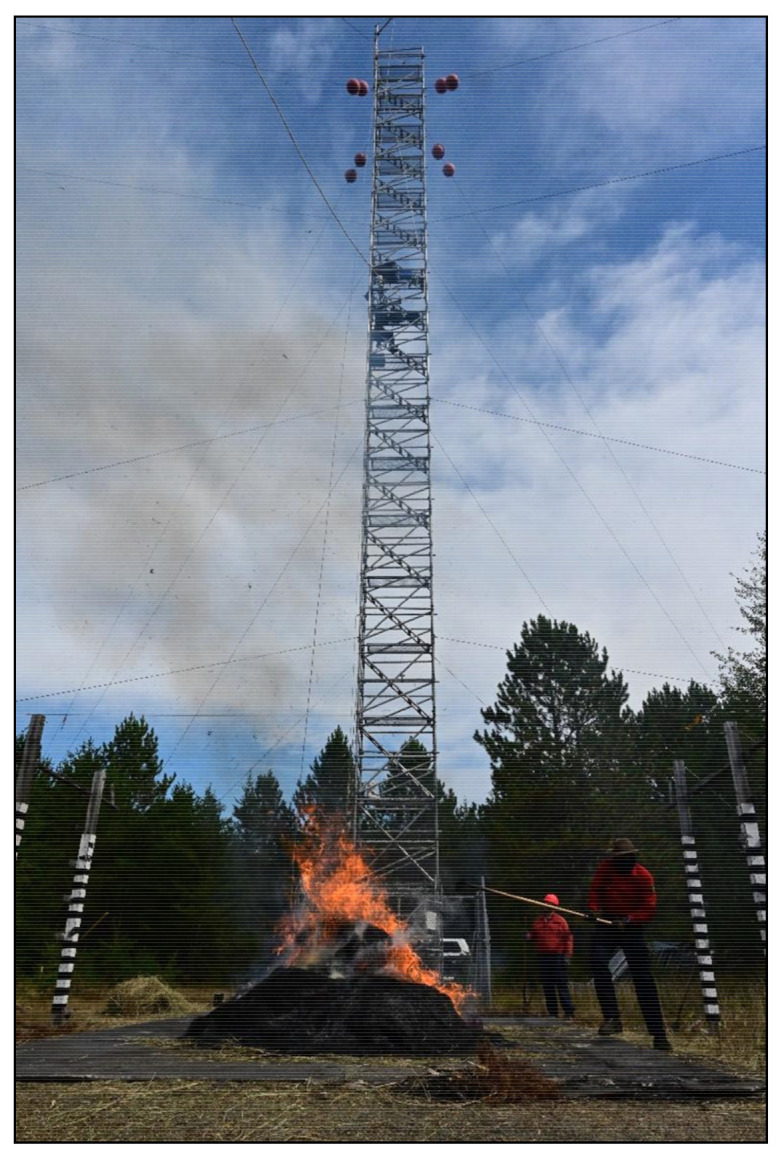
Example of experimental burn trials conducted 18 September 2019 during this experiment. Pictured here, researchers add fuel to a ~2 m diameter fire with a pitchfork while it is sampled by Bomberos and the reference FLIR imager on the tower adjacent to the burn platform.

Once imaging had begun, a series of experimental fires were lit on the burn platform using recently harvested hay (primarily Timothy grass; *Phleum pratense*) from nearby agricultural activities. Although not a forest fuel itself, there is precedence for its use in FRP studies [21]. The experimental fires themselves ranged in size from 0.25 to 4 m in diameter and, when smaller diameters were burning, multiple fires would be spaced throughout the platform in varying spatial configurations (Figure 3). Throughout the burning process, fires were allowed to smolder down and be stoked up by the periodic addition of more fuel, resulting in longer durations for sampling and a broad range of intensities.

### 2.3. Data Processing and Analysis

#### 2.3.1. Temporal and Spatial Characterization

Temporal alignment between the Bomberos and FLIR images was performed prior to calculating FRP for each image. The Bomberos images were recorded in one-second intervals, whereas the FLIR images were recorded continuously throughout the burns, and, therefore, the FLIR images had to be segmented to match the Bomberos sequences. Time synchronization was done by visually examining the images of the Bomberos and FLIR data to look at commonalities between the images (e.g., people appearing in the frames, more fuel being added to the fire). Once the images were matched visually, they were checked by creating a time series of the mean FRP for frame and ensuring that the images were comparable.

Spatial characterization between the Bomberos and FLIR images was determined by using ambient images from both cameras and known measurements of burn pad panels to calculate the approximate pixel size for each of the Bomberos and FLIR image outputs. Measurements were taken from four different locations across the burn pad to produce four different pixel sizes for each image. These four pixel sizes were then averaged to produce one pixel size for each of the Bomberos and FLIR images. New measurements were taken every time the camera angle changed to ensure that the new position of the camera angle did not affect the pixel size. On average, the FLIR pixel size was 0.025 m in diameter and the Bomberos was 0.022 m. These approximate pixel sizes for each of the Bomberos and FLIR images were used in the FRP calculation.

#### 2.3.2. Fire Radiative Power Calculations

Fire radiative power was calculated separately for both the MWIR and LWIR bands. For the Bomberos MWIR and FLIR MWIR FRP calculations, the MWIR radiance method of Wooster et al. [20] was used. The FRP calculation coefficient was tailored to the specific spectral response functions of the Bomberos and FLIR cameras. Before calculating FRP, all pixels with a temperature of less than 500 K were removed from the images. This was to remove pixels that were detected in the Bomberos imagery at a lower threshold than the FLIR imagery, ensuring that the datasets were comparable. For the Bomberos LWIR camera, FRP was calculated from the brightness temperature with an assumption that the fire behaved as a blackbody, filling the entirety of each pixel area, allowing FRP to be calculated using the Stefan–Boltzmann law. For both the MWIR and LWIR FRP calculations, FRP was calculated for each pixel in the image, and then summed to produce total FRP per frame for the fire in kilowatts (kW).

#### 2.3.3. Bomberos MWIR—FLIR MWIR Intercomparison

The Bomberos MWIR and FLIR MWIR intercomparison for FRP was carried out by comparing the mean FRP for each sequence. The sum of FRP per frame was taken for both the Bomberos MWIR and FLIR MWIR data, and then the mean was calculated for each one-second sequence. The field of view for the Bomberos and FLIR cameras were not the same, so in instances where there were multiple fires on the burn pad, each fire was isolated in both the Bomberos and FLIR images so that the FRP comparison would only be for a single fire. In cases where there was only one fire on the burn pad, the entire field of view for each camera was compared in the FRP comparison. The mean FRP values for each sequence of the Bomberos MWIR and FLIR MWR data were then plotted against each other and compared using linear regression with the intercept constrained at 0. In this analysis, the slope from the linear regression estimated the deviance of the FRP values from the line of perfect agreement. A separate *t*-test was also conducted to determine if the slope of the relationship between the FLIR MWIR and Bomberos MWIR FRP data was significantly different from the line of perfect agreement. There were 67 one-second fire sequences, resulting in one mean FRP estimation each, which were compared in this analysis.

#### 2.3.4. Bomberos MWIR—LWIR Intercomparison

The Bomberos MWIR and LWIR intercomparison was analyzed using the same methods described above. A linear regression model with the intercept constrained at zero was used and results were analyzed to determine the deviance of the MWIR and LWIR FRP values from the line of perfect agreement. Similarly to the Bomberos and FLIR MWIR intercomparison, a *t*-test was conducted to determine if the slope of the relationship between Bomberos MWIR and LWIR was significantly different from the line of perfect agreement. There were 67 fire sequences compared in this analysis.

## 3. Results

### 3.1. Bomberos MWIR and FLIR MWIR FRP Comparison

**Figure 4:**
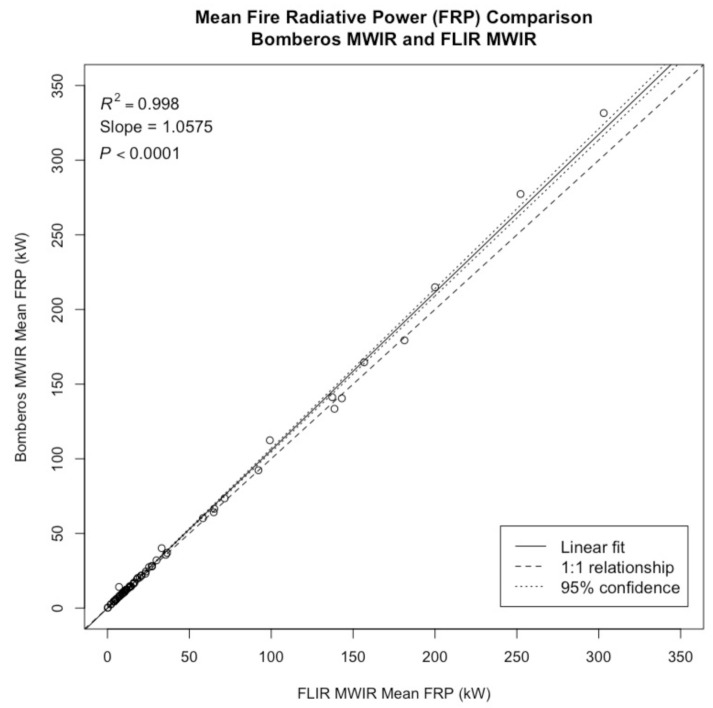
Linear regression between the FLIR MWIR and Bomberos MWIR fire radiative power (FRP). Data points consist of 67 different one-second sequences recorded from experimental fires conducted between 16 and 18 September 2019.

**Figure 5:**
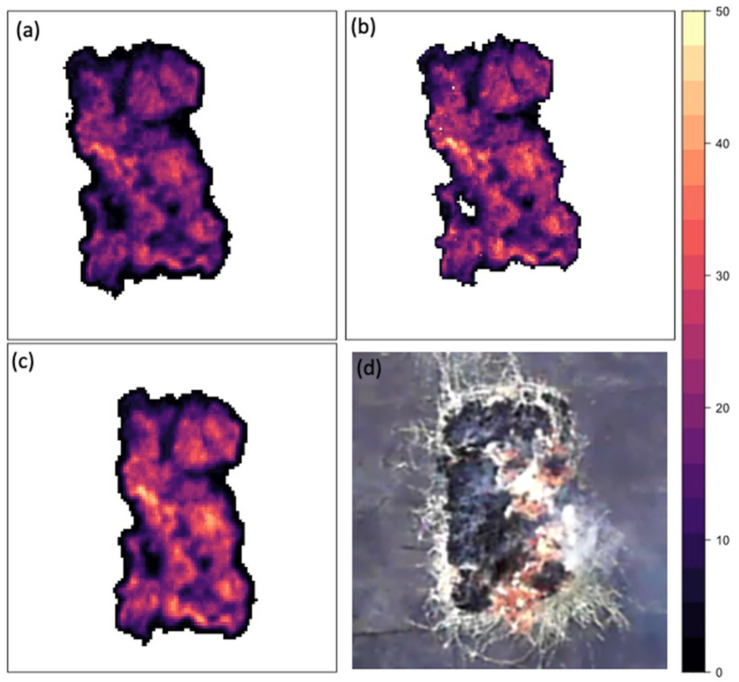
Images of a 1.5 × 3 m fire on 16 September 2019. (**a**) MWIR Bomberos image of FRP per pixel in Watts. The total FRP for the fire in the frame is 64,236.2 W (64.3 kW); (**b**) LWIR Bomberos image of FRP per pixel in Watts. The total FRP for the fire in the frame is 72,651.0 W (72.7 kW); (**c**) MWIR FLIR image of FRP per pixel in Watts. The total FRP for the fire in the frame is 63,642.9 W (63.6 kW); (**d**) Visual imagery of the fire from a camera fixed to a tower 21.6 metres above the fire.

The linear regression between the Bomberos MWIR and FLIR MWIR FRP shows a significant relationship (R^2^ = 0.998, *p* < 0.0001; see Figure 4). Most data points follow the line of perfect agreement. Overall, there was more data collected at lower FRP values than higher FRP values. The agreement between FRP values from Bomberos and FLIR is extremely good for lower FRP values; however, as FRP increases, there is deviation from the line of perfect agreement, with Bomberos estimating slightly higher FRP values than FLIR. The slope of the relationship between Bomberos MWIR and FLIR MWIR is 1.06, meaning that there is a 6% deviance between the FLIR MWIR and Bomberos MWIR relationship and the line of perfect agreement. This difference between the slope and the line of perfect agreement is significant, t(66) = 9.31, *p* < 0.001. Figure 5a,c show the FRP values per pixel for the Bomberos MWIR and FLIR MWIR images, respectively, for an example frame in a sequence, with the two images being very comparable in pixel FRP values and overall mean FRP.

### 3.2. Bomberos MWIR and LWIR FRP Comparison

**Figure 6:**
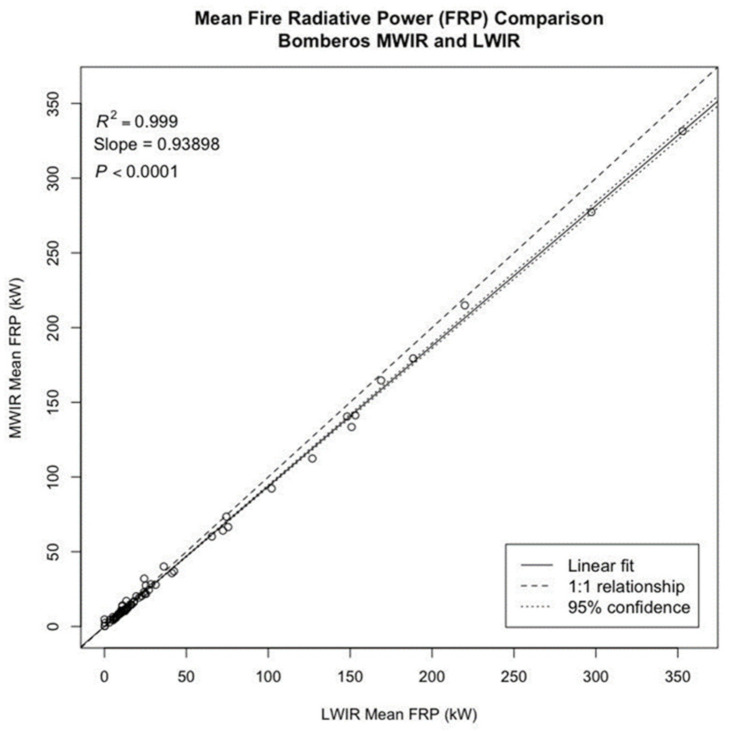
Linear regression between the Bomberos MWIR and LWIR fire radiative power (FRP). Data points consist of 67 different one-second sequences recorded from experimental fires conducted between 16 and 18 September 2019.

The linear regression between the Bomberos MWIR and LWIR FRP shows a significant relationship (R^2^ = 0.999, *p* < 0.0001; see Figure 6). The mean FRP for the Bomberos MWIR and LWIR align closely with the line of perfect agreement, with deviation from the 1:1 line increasing as FRP increases. At higher values of FRP, the Bomberos LWIR estimates higher FRP values than the Bomberos MWIR. The slope of the relationship between the Bomberos MWIR and LWIR mean FRP, 0.94, is significantly different from the line of perfect agreement, t(66) = −14.21, *p* < 0.01.

## 4. Discussion

This study examines the use of microbolometer-based mid- and long-wave IR detectors for collecting radiometric measurements from biomass combustion. Through direct comparison between measurements made with the Bomberos microbolometer test bench and coincident FLIR imagery of 67 experimental burning samples, it was determined that the microbolometer detectors were highly reliable in retrieving FRP in both the mid- and long-wave IR. That said, in both spectral bands, the relationship between Bomberos and FLIR FRP did show statistically significant deviation from the line of perfect agreement.

Regression analysis of the Bomberos and FLIR MWIR FRP retrievals demonstrate exceptional correlation (R^2^ = 0.998, *p* < 0.0001), though the slope from this comparison, 1.06, is a small but statistically significant deviation from the line of perfect agreement (t(66) = 9.31, *p* < 0.001). This trend towards over-estimation (of ~6%) by Bomberos is more pronounced at higher FRP and may be explained by a number of potential factors. Notably, the FLIR and Bomberos spatial resolutions differed (~0.022 m and ~0.025 m diameter for Bomberos and FLIR, respectively) due to their detector dimensions and optical configurations. Given that pixel area corrections were estimated manually in the analysis, it is possible that minor errors in these calculations could have impacted the accuracy of either the FLIR or Bomberos FRP calculations. Additionally, the temporal co-registration of the imagery is not exact, which could have influenced the comparison; however, the comparison of mean total FRP over the sample periods was designed to minimize these errors. Though the two cameras have different saturation points, in both data sets, saturation was not observed. Aside from the fundamental difference in detector technology, the most significant difference between the Bomberos and FLIR MWIR detectors is their spectral response functions. While the FLIR system was equipped with a narrow pass (~0.15 µm) filter that stops short of 4.0 µm, the Bomberos MWIR bandpass filter is much wider, extending from 3.4 to 4.2 µm and potentially intersecting the CO_2_ band around 4.2 µm. Given that biomass combustion releases large amounts of hot CO_2_ (which varies substantially throughout the vertical profile of the smoke plume), and since the absorption of ambient CO_2_ is a strong factor even in short-range measurements, even a small deviation within this spectral range could introduce substantial differences in the FRP observations. It should also be noted that the lower temperature threshold for the data segmentation was determined based on the brightness temperature ranges, and not modified to account for the varying spectral response functions of the two cameras; however, the net effect of this discrepancy is minimal on overall FRP calculations. Notably, despite these potential causes, the agreement between the FRP observations remains strong.

Regression analysis of the Bomberos MWIR and LWIR FRP retrievals demonstrate similarly strong correlation (R^2^ = 0.999, *p* < 0.0001) but with a slope of 0.94, which is again a statistically significant deviation from the line of perfect agreement (t(66) = −14.21, *p* < 0.01). In this case, the trend is towards over-estimation (of ~6%) in the LWIR. Though the bias is small, here, the over-estimation is more consistently observed across the full range of observations. It should be noted that not only are the MWIR and LWIR observations being recorded in completely different spectral bands, but they are also calculating FRP in very different ways. Based on the variation in the calculation method (MWIR radiance method vs. Stefan–Boltzmann law), disagreement in the results is to be expected. With this in mind, the agreement between the MWIR and LWIR FRP retrievals is considered very successful.

## 5. Conclusions

Early efforts to image biomass burning events using MWIR microbolometer technology demonstrated that it was possible to detect these phenomena from space-borne platforms (e.g., [29,33]). However, this demonstration failed to include complete radiometric calibration capabilities, resulting in the inability to derive FRP from the data. As a result, some debate has persisted within the community as to the applicability of MWIR microbolometer technology in providing comprehensive fire monitoring datasets. In this study, we have demonstrated that it is possible to perform high-accuracy radiometric observations of biomass burning events in the MWIR and LWIR using microbolometer technology. To our knowledge, this study represents the first scientific evidence that MWIR microbolometers are highly reliable sources of FRP measurements. In doing so, these findings suggest that these types of low resource detectors are suitable for deployment for wildfire monitoring applications, particularly where onboard resources are limited (e.g., UAV and nanosat type scenarios).

The encouraging results from this study are expected to stimulate further developments towards a compact system specifically designed for radiometric airborne and space-based wildfire measurement. This could include combining MWIR and LWIR bands in a single camera—for example, by using butcher block filters near the focal plane array, or with a filter wheel. Moreover, the development of a less resource-intensive thermal offset compensation method would likely be required. This could potentially be achieved by using a lightweight mechanical shutter, or by using temperature measurements at strategic points in the camera and correcting the images accordingly.

## Data Availability

The data presented in this study are available on request from the corresponding author.
